# Different Infective Forms Trigger Distinct Immune Response in Experimental Chagas Disease

**DOI:** 10.1371/journal.pone.0032912

**Published:** 2012-03-07

**Authors:** Paula Melo de Abreu Vieira, Amanda Fortes Francisco, Evandro Marques de Meneses Machado, Nívia Carolina Nogueira, Kátia da Silva Fonseca, Alexandre Barbosa Reis, Andrea Teixeira-Carvalho, Olindo Assis Martins-Filho, Washington Luiz Tafuri, Cláudia Martins Carneiro

**Affiliations:** 1 Laboratório de Imunopatologia, Núcleo de Pesquisas em Ciências Biológicas (NUPEB), Universidade Federal de Ouro Preto (UFOP), Ouro Preto, Minas Gerais, Brazil; 2 Laboratório de Doença de Chagas, Núcleo de Pesquisas em Ciências Biológicas (NUPEB), Universidade Federal de Ouro Preto (UFOP), Ouro Preto, Minas Gerais, Brazil; 3 Laboratório de Biomarcadores de Diagnóstico e Monitoração, Centro de Pesquisas Rene Rachou, FIOCRUZ, Belo Horizonte, Minas Gerais, Brazil; 4 Departamento de Análises Clínicas, Escola de Farmácia, Universidade Federal de Ouro Preto (UFOP), Ouro Preto, Minas Gerais, Brazil; University of South Alabama, United States of America

## Abstract

Although metacyclic and blood trypomastigotes are completely functional in relation to parasite-host interaction and/or target cell invasion, they differ in the molecules present on the surface. Thus, aspects related to the variability that the forms of *T. cruzi* interacts with host cells may lead to fundamental implications on the immune response against this parasite and, consequently, the clinical evolution of Chagas disease. We have shown that BT infected mice presented higher levels of parasitemia during all the acute phase of infection. Moreover, the infection with either MT or BT forms resulted in increased levels of total leukocytes, monocytes and lymphocytes, specifically later for MT and earlier for BT. The infection with BT forms presented earlier production of proinflammatory cytokine TNF-α and later of IFN-γ by both T cells subpopulations. This event was accompanied by an early cardiac inflammation with an exacerbation of this process at the end of the acute phase. On the other hand, infection with MT forms result in an early production of IFN-γ, with subsequent control in the production of this cytokine by IL-10, which provided to these animals an immunomodulatory profile in the end of the acute phase. These results are in agreement with what was found for cardiac inflammation where animals infected with MT forms showed intense cardiac inflammation later at infection, with a decrease in the same at the end of this phase. In summary, our findings emphasize the importance of taking into account the inoculums source of *T. cruzi*, since vectorial or transfusional routes of *T. cruzi* infection may trigger distinct parasite-host interactions during the acute phase that may influence relevant biological aspects of chronic Chagas disease.

## Introduction

The protozoan, *Trypanosoma cruzi*, is the etiological agent of American trypanosomiasis, known as Chagas disease, which is a major public health problem in South and Central America. The protozoan exists in at least three morphologically distinct stages. Epimastigotes proliferate in the invertebrate host and are released as metacyclic trypomastigotes (MT) in the faeces. In the vertebrate host, amastigotes and blood trypomastigotes (BT) are the intracellular developmental and infective forms, respectively [1]. Vectorial infection is the main and most frequent form of transmission that occurs when mucous membranes or abraded skin are exposed to MT-infected faeces of triatomine insects [2]. However, transmission by blood transfusion is also an important route of transmission, because BT forms remain viable in blood products stored in blood banks, especially in non-endemic areas, such as Europe, where there is no control for this disease in blood banks [3]. In the United States, the routine screening of blood donors for *T. cruzi* infection has been initiated in January 2007 and covers 75–90% of the blood supply [4].

Although metacyclic and blood trypomastigotes are completely functional in relation to parasite-host interaction and/or target cell invasion [5], they differ in the molecules present on the surface. Studies have shown that glycosyl phosphatidylinositol-anchored mucin-like glycoproteins purified from BT forms (tGPI mucins) are potent elicitors of proinflammatory responses (i.e. cytokine and NO production) by IFN-γ primed murine macrophages. In contrast, the corresponding glycoproteins derived from MT forms (mGPI mucins) are reported to be less active than tGPI mucins in the induction of NO by murine macrophages [6] [7] [8]. Moreover, it is known that some glyco-inositol-phospholipids (GIPLs), extracted from the cell membrane of MT forms, exert suppressive function in the activation of macrophages and dendritic cells, inhibiting the secretion of TNF-α and IL-12 [9]. We have reported that BT and MT infections in dogs are associated with distinct parasitological and serological features, together with intrinsic and inoculum source-specific changes in circulating leukocytes [10] [11]. The results suggest that the source of inoculum can interfere with the development of the acute phase of Chagas disease, and may also trigger a distinct parasite-host interaction during this phase.

Cytokines play an important role in controlling the replication of the parasite and the immune response in infected animals. In Chagas disease, inflammatory cytokines are essential during the acute phase of infection, being produced at high levels in the chronic phase, possibly by chronic exposure to the parasite [12], [13] [14]. The high expression of proinflammatory cytokines, especially IFN-γ and TNF-α, has been associated with the progression of severity in cardiac injury [15] [16]. However [17], found a negative correlation between the expression of IFN-γ and cardiomyopathy. Thus, the involvement of IFN-γ in the development of cardiac lesions remains controversial. Moreover, Souza *et al.* (2004) [18] showed that monocytes from patients with indeterminate form have a higher expression of IL-10 after exposure to the parasite, whereas monocytes from patients undergoing cardiac form to the same treatment preferentially express TNF-α. Other authors have also observed high expression of IL-10 by cells from patients with the indeterminate form [19]. Thus we can speculate that individuals who remain asymptomatic can reduce the number of parasites at the beginning of infection, regulating the immune response in a way that limits the development of lesions. Moreover, although individuals who go on to develop the cardiac form can control the parasites, they are unable to mount an immunoregulatory response, which leads to persistent inflammation.

In this sense, the study of infection in mice by different infective forms of *T. cruzi* during the acute phase of infection will enable a better understanding of the mechanisms related to the pathogenesis of Chagas disease. Moreover, due to the fact that the transfusion and congenital infection in non-endemic countries is recognized as a serious problem, it is important to know what the impact of infection by BT forms in the course of the disease. Based on this, the main goal of the current work was to investigate kinetically alterations in parasitemia and leukocytes of the peripheral blood, cardiac inflammation and the cytokine profile of T-cell subsets in the spleen during the acute-phase of experimental infection by MT forms, simulating vectorial transmission, and by BT forms, simulating transfusion transmission (or, indeed, any transmission mechanism involving BT forms) of *T. cruzi*.

## Results

### BT infection promoted higher levels of parasitemia

**Figure 1:**
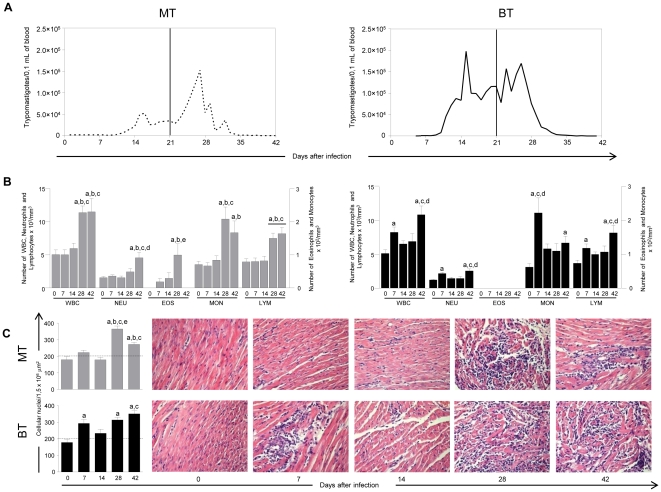
Kinetics of parasitemia, leukocytes in the peripheral blood and heart inflammatory infiltrate before infection (0) and at 7, 14, 28 and 42 days after mice infection with metacyclic (MT; light gray bar) or blood trypomastigotes (BT; black bar) of *Trypanosoma cruzi*. (**A**) Levels of Parasitemia during the acute phase. (**B**) Kinetics of the Leukocytes subsets in the peripheral blood. Leukocytes subsets were identified by cell smear counting as described in Material and Methods (**C**) Morphometric analysis and photomicrographs of heart showing an intense inflammatory infiltrate starting at 7^th^ and 28^th^ after infection in the animals of the group BT and MT, respectively. The results are expressed as mean number of cells ± standard error. Significant differences at p<0.05 are highlighted by letters “a”, “b”, “c”, “d” and “e” for comparisons with Day 0, Day 7, Day 14, Day 28 and Day 42, respectively.

Infection was confirmed in all mice that had been inoculated with MT or BT forms of Be-78 *T. cruzi* strain, although mortality was not observed within the 42-day experimental period. The kinetic of the parasitemia curves are shown in Fig. 1A. Animals infected with BT forms presented higher levels of parasitemia during the 42 days of assessment.

### MT infection triggers a delayed alteration on blood leukocytes

To investigate whether the infection by MT or BT forms could interfere with the circulating leukocytes in the blood, differential counts were done on these cells, and the results of blood cell counts within the period immediately before inoculation and for the following 42 days are shown in Fig. 1B. Infection with either MT or BT forms resulted in significantly increased levels of White Blood Cells (WBC), monocytes and lymphocytes, specifically on days 28 and 42 for MT and on days 7 and 42 for BT. Neutrophils showed a significant increase only at 42 days in animals infected with MT forms, but this increase occurred early in the 7^th^ day for animals infected with BT forms and further increased again by the 42^th^ day. In animals infected with MT forms, eosinophils were significantly elevated by the 28^th^ day, but there was no significant increase for animals infected with BT forms in the number of these cells over 42 days.

### BT infection showed an early cardiac inflammation that remains intense over 42 after infection

Morphometric analyses of inflammatory infiltrate in the heart are given in Fig. 1C. Statistically significant increases were observed in the number of cell nuclei present in the heart fragments from both MT and BT infected animals. In the BT group, the increase of inflammatory infiltrate occurred at 7^th^, 28^th^ and 42^th^ day whereas in the MT group a significant increase was seen at days 28^th^ and 42^th^ as comparison to the baseline. However, it was interesting to notice that the number of inflammatory cells decreased significantly on 42^th^ day as compared to day 28^th^ in the MT group. In all infected animals, the inflammatory infiltrate was predominantly of mononuclear cells (Fig. 1C), the majority of which had lymphocyte morphology.

### MT infection presents isolated amastigotes in the heart while BT infection is characterized by the presence of typical amastigote nests

**Figure 2:**
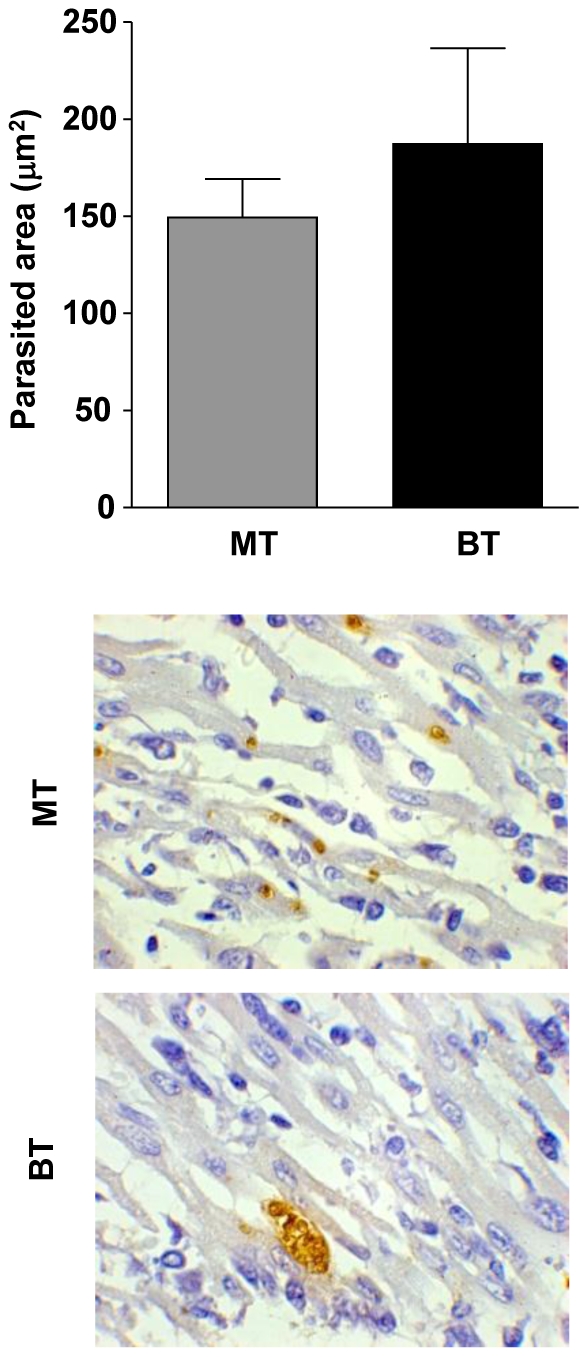
Morphometric analysis and photomicrographs of the area of *T. cruzi* immunoreactions in the heart at 28 days after mice infection with metacyclic (MT; light gray bar) or blood trypomastigotes (BT; black bar) of *Trypanosoma cruzi*. Analysis of the area of *T. cruzi* amastigotes were identified by immunohistochemistry as described in Material and Methods. The results are expressed as parasited area ± standard error. Photomicrographs of the heart parasitism demonstrating isolated amastigotes in MT and typical amastigote nests in BT.

In order to quantify the heart parasitism an anti-*T. cruzi* immunohistochemistry was performed. There was no isolated amastigote or typical nests on days 7, 14 and 42 after infection. Only at day 28 after infection was observed isolated amastigotes in MT and typical amastigote nests in BT, however no significant difference between the two experimental groups was observed on this day (Fig. 2).

### Although MT and BT infections lead to increased percentage of CD8^+^ T splenocytes, BT elicited early increase in CD8^+^ T-cell-derived TNF-α and IL-10

**Figure 3:**
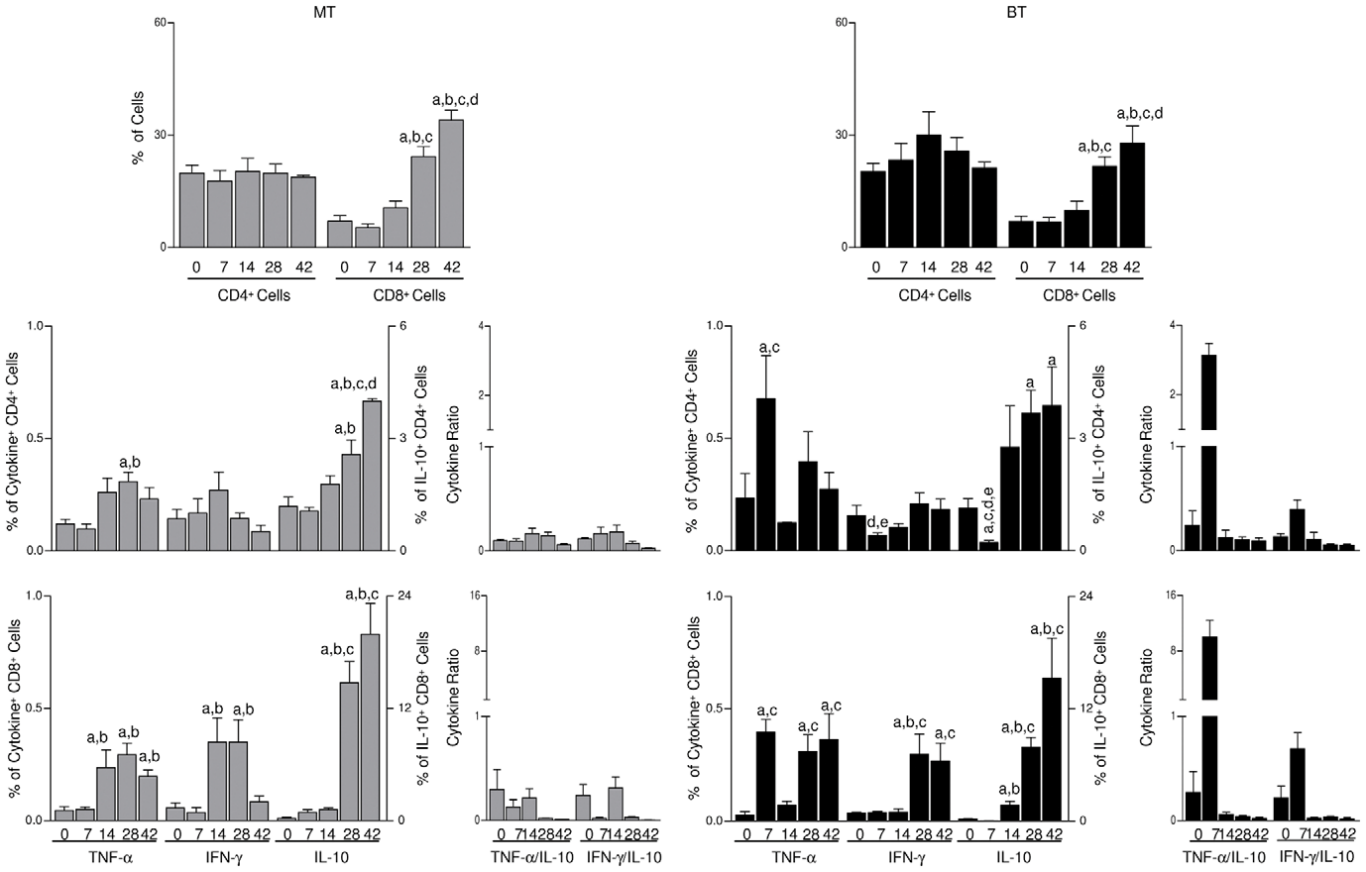
Immunophenotypic profile and cytokine pattern (TNF-α, INF-γ and IL-10) of CD4^+^ and CD8^+^ T-splenocyte before infection (0) and at 7, 14, 28 and 42 days after mice infection with metacyclic (MT; light gray bar) or blood trypomastigotes (BT; black bar) of *Trypanosoma cruzi*. (**A**) Analysis T-cells subsets were identified by flow cytometric immunostaining as described in Material and Methods. Data were expressed as percentage of positive cells within gated lymphocytes. (**B**) Splenocytes were cultured *in vitro* in the absence of antigen. TNF-α^+^, IFN-γ^+^ and IL-10^+^ CD4^+^ and CD8^+^ were analyzed after flow cytometric immunostaining for cell surface markers and intracellular cytokines. The results are expressed as mean percentage of cytokine+cells ± standard error. Significant differences at p<0.05 are highlighted by letters “a”, “b”, “c”, “d” and “e” for comparisons with Day 0, Day 7, Day 14, Day 28 and Day 42, respectively.

Aiming to characterize the effect of infection with MT or BT forms in lymphocyte subpopulation, we performed immunophenotypical analysis of blood and spleen cells and also characterized the cytokine pattern of T splenocytes. The phenotypic profiles of blood T lymphocytes subpopulations show that BT lead to a slight decrease in both CD4^+^ and CD8^+^ T cells at day 7^th^ after infection (17% and 21% as compared to 34% and 36% at baseline, respectively) with no changes observed in the MT group (data not shown). Regardless of the infective form, the infection with *T. cruzi* does not alter the percentage of splenic CD4^+^ T cells. However, an increase in the percentage of splenic CD8^+^ T cells was observed in both experimental groups (Fig. 3).

In attempts to focus more deeply on the impact of MT or BT infection on the T-cell cytokine pattern, we have characterized the frequency of TNF-α^+^, IFN-γ^+^ and IL-10^+^ T-cells as their major subsets (CD4^+^ and CD8^+^) within splenocytes. The frequency of TNF-α^+^, IFN-γ^+^ and IL-10^+^ T-cell subsets in the cultures are shown in Fig. 3.

Regarding TNF-α, infection by MT and BT forms triggered different patterns; in the MT group there is a significant increase at days 14, 28 and 42 by CD8^+^ T cells and on day 28 by CD4^+^ T cells. In the BT group the synthesis of TNF-α by T-cells was significantly increased at day 7, rising again at days 28 and 42 for CD8^+^ T cells; this demonstrates that there is a preferred production of TNF-α by T cells in infection by BT forms.

No changes were observed in IFN-γ synthesis by CD4^+^ T-cells in the MT group, but there were a significant decrease at day 7 in the BT group. Significantly increased percentages of CD8^+^IFN-γ^+^ T-cells were observed in MT and BT groups on days 14 and 28 for MT, but only on day 28 for BT, demonstrating an earlier increase in the levels of IFN-γ by CD8^+^ T cells in the MT group.

The BT group showed a decrease in the percentage of cells CD4^+^IL-10^+^ at day 7 after infection, however higher levels of IL-10^+^ T-cells were seen in both groups, but this level increased at day 28 in MT group, an effects seen this increase occurred earlier at day 14 in the BT group.

### Despite triggering distinct cytokine profiles, MT forms shifted the overall cytokine balance toward a type-1 immune response

**Figure 4:**
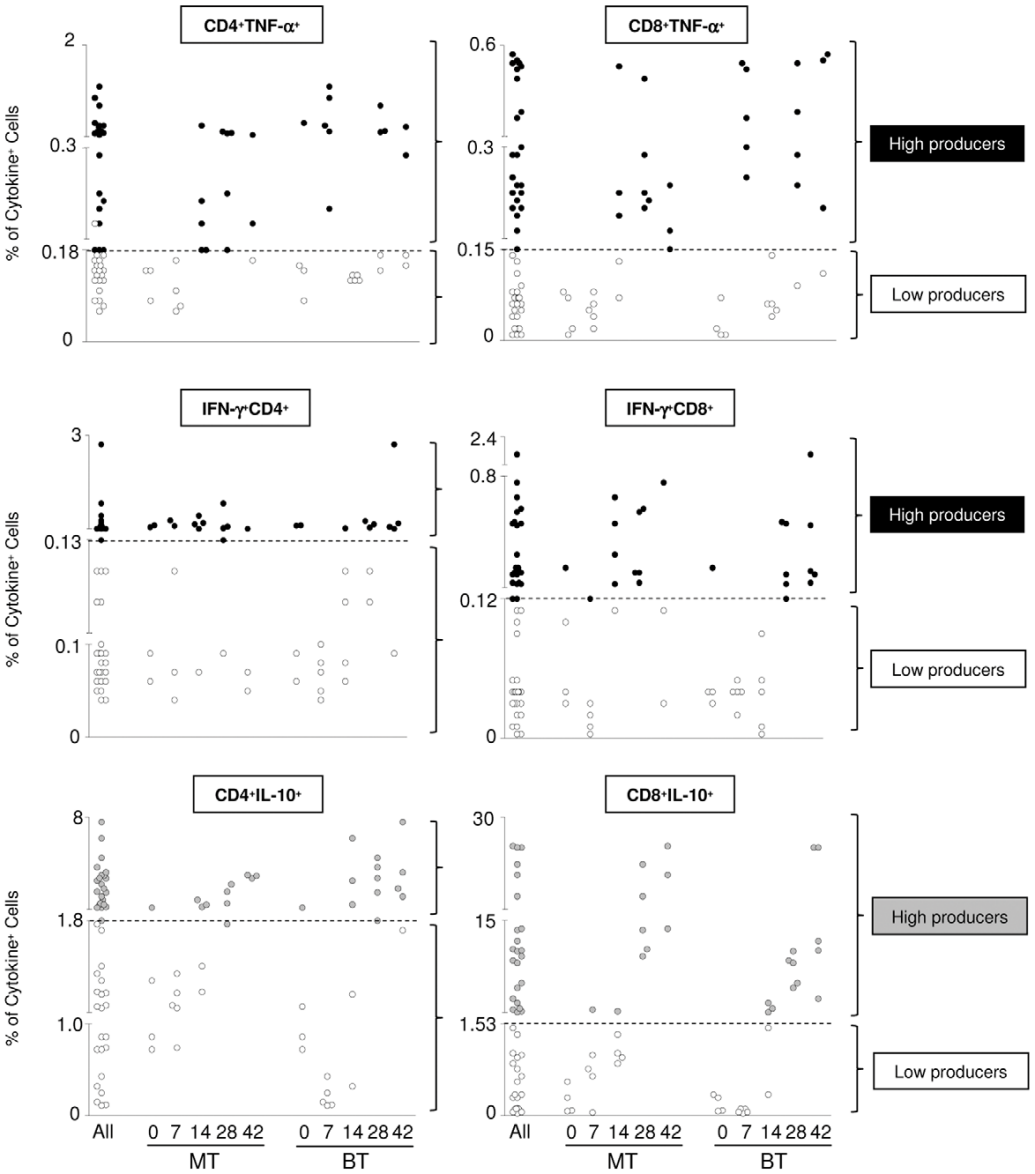
Representative scatter graphs employed to identify “low” cytokine-producers (White circle), “high” TNF-α or IFN-γ producers (black circle) and “high” IL-10-producers (light gray circle) amongst spleen T-lymphocytes from mice before infection (0) and at 7, 14, 28 and 42 days after infection with metacyclic (MT) or blood trypomastigotes (BT) of *Trypanosoma cruzi.* * “Low” and* “high” cytokine-producers were defined for each T- lymphocyte subset based on global median cut-off edge (- - -) obtained for the whole study population (all mice). Distinct cut-offs were employed for TNF-α^+^CD4^+^, IFN-γ^+^CD4^+^, IL-10^+^CD4^+^ T-cells, TNF-α^+^CD8^+^, IFN-γ^+^CD8^+^ and IL-10^+^CD8^+^ T-cells.

**Figure 5:**
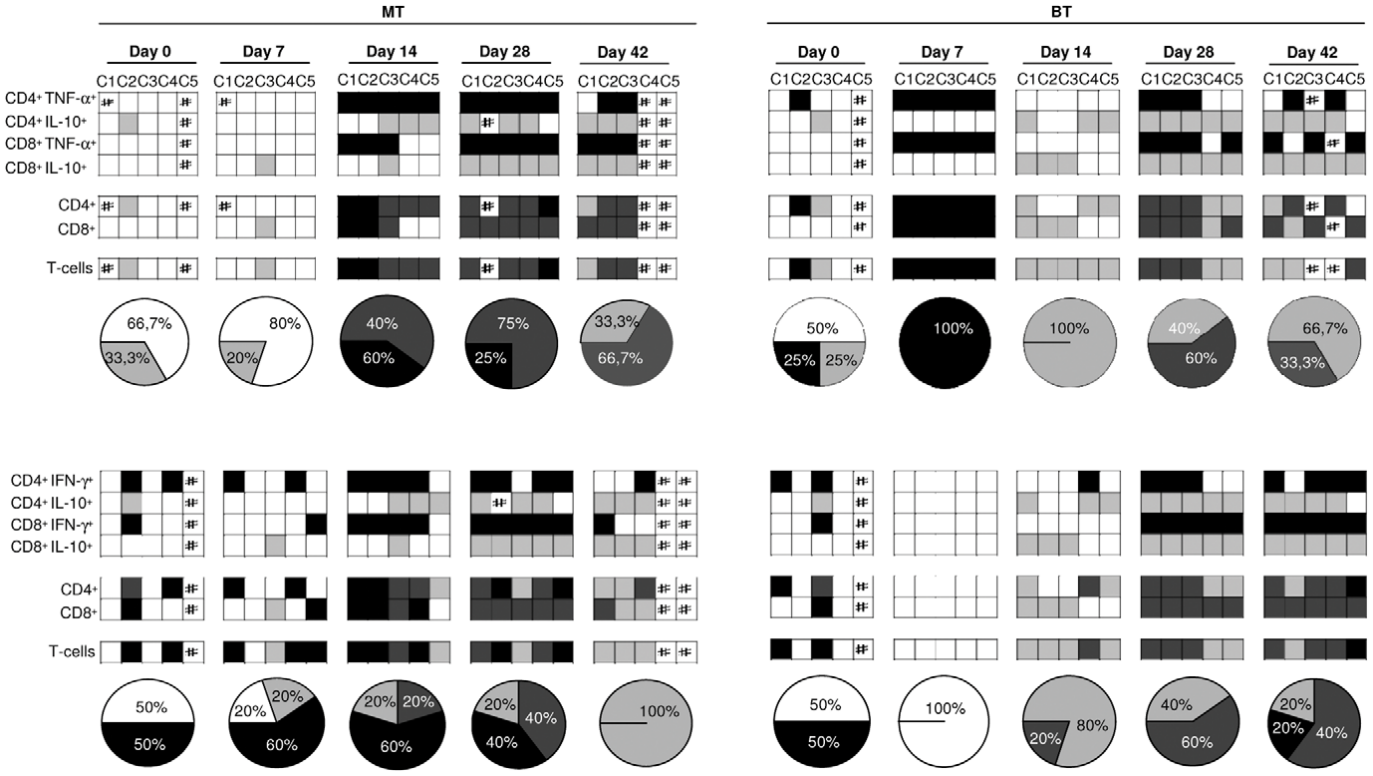
Cytokine profile of spleen T-lymphocytes from mice before (0) and at 7, 14, 28 and 42 days after infection with metacyclic (MT) or blood trypomastigotes (BT) of *Trypanosoma cruzi*. “Gray scale” diagrams were used to represent the cytokine pattern and the cytokine balance within T-cell subsets besides the overall cytokine balance with T-cells, highlighting the predominance of “low” cytokine-producers (white square), “high” TNF-α or IFN-γ producers (black square), “high” IL-10^+^ producers (light gray square) or “high” mixed cytokine-producers (dark gray square). Pie charts represent the percentage of animals displaying a given T-cells overall cytokine balance selectively amongst the “high” cytokine-producers. ND = Not detected.

Taking the general hypothesis that a fine balance between pro-inflammatory cytokines (IFN-γ and TNF-α) and IL-10 profile is more relevant than a shift toward a polarized cytokine pattern, we characterized for each animal within the MT and BT groups their overall balance of IFN-γ/IL-10 and TNF-α/IL-10 derived from CD4^+^ and CD8^+^ T-cell subsets. This strategy allows the characterization of the resultant cytokine profile from T-cells driven by infection due to different infective forms. To establish the overall cytokine balance data from flow cytometry, the results were further transformed as proposed by Vitelli-Avelar *et al.* [20]. This new strategy consists of a 4-step platform that includes: (i) establishment of “low” and “high” cytokine-producers based on global median of cytokine^+^ T-cell subsets calculated from the whole range of values obtained for the entire study population (all mice) including MT or BT infected mice (Fig. 4); (ii) construction of “gray scale” diagrams for each group of animals (MT and BT group) showing the “*cytokine pattern*” of “low” and “high” cytokine-producers within CD4^+^ and CD8^+^ T-cell subsets (Figs. 4 and 5); (iii) compilation of the “*cytokine balance*” defined as predominant low cytokine producers, inflammatory, regulatory or mixed cytokine-producers within T-cells (Fig. 5) and (iv) assembly of the “*overall cytokine balance*” as the proportion of low cytokine; high inflammatory, regulatory or mixed cytokine producers within T-cells (Fig. 5).

The analysis of the “*cytokine pattern*” of T-cell subsets, based on the 3 major classes of cytokine-producers named as “low” cytokine-producers, “high” TNF-α and IFN-γ producers and “high” IL-10 producers, observed in “gray scale” diagram among MT mice, showed that there was a predominance of “high” cytokine-producers within all T-cell subsets at days 14 and 28 compared to day 0 (Figs. 4 and 5). While for the BT group, all the mice showed a pattern of “low” cytokine-producers at day 7, with a predominance of “high” cytokine-producers within all T-cell subsets only at days 28 and 42 compared to day 0 (Figs. 4 and 5), thereby showing delayed production of the cytokines in this group.

Taking the “*cytokine balance*” within the CD4^+^ and CD8^+^ T-cell subsets, our data demonstrate the predominant pattern of a distinct cytokine balance in the MT and BT groups, with a distinct predominance of animals displaying inflammatory or mixed cytokine profiling, respectively (Fig. 5).

Analysis of the “*overall cytokine balance*” also showed that the MT and BT forms induced the pattern of “high” cytokine-producers in all infected mice from day 14 onwards (Fig. 5). Indeed, the MT form drove a predominant IFN-γ profile until day 28, suggesting an inflammatory pattern (Fig. 5, pie chart). But the MT group was capable to shift the overall cytokine profile toward a regulatory profile at day 42. On the other hand, the BT form drove a predominant IL-10 profile or a mixed IFN-γ ≈IL-10 pattern at day 14 onwards (Fig. 5, pie chart). And had an inflammatory profile on the seventh day after infection to be held the balance between cytokines TNF-α/IL-10.

## Discussion

This paper reports a follow-up investigation of the major immunological features in mice experimentally infected with metacyclic or blood forms of *T. cruzi*. The two infective forms of the parasite are known to exhibit distinct characteristics that induce different immune responses *in vitro* [21]. Infection mediated by metacyclic forms *in vitro* can, in part, mimic that caused by vectors *in vivo*. On the other hand, *in vivo* infection with blood forms imitates blood and congenital transmission or even laboratory accidents. It is worth mentioning that the majority of experimental infections are conducted using blood trypomastigotes because these forms are easier to obtain and maintain in the laboratory. Moreover, the recent increase in cases of blood transmission in non-endemic countries is relevant to understanding the host-parasite interaction in these conditions, compared to infection by MT forms.

Shifts of both the prepatent period and the day of maximum parasitemia, respectively, from day 6 and 15 in BT and from day 8 and 27 in MT, were observed. The longer prepatent period, together with the later parasitemia peak, in MT-infected animals suggested that parasite/host cell interaction may differ depending on the inoculums source. Similar results were obtained previously by our group in dogs infected with Be-78 strain of *T. cruzi* [10], suggesting a differential impact of the inoculums source on the development of the acute phase of Chagas disease. These data may be related to the fact that BT forms, but not MT, are typically associated with membrane-bound anti-*T. cruzi* immunoglobulins, which could facilitate the entry of this form in a greater number of target cell thus leading to earlier parasite-host cell events interaction favoring their faster circulation release into the system of the host. These results corroborate with heart parasitism data showing isolated amastigotes in MT and typical amastigote nests in BT, demonstrating a rapid proliferation of the parasite by BT forms.

Acute infection in mice leads to strong activation of innate and adaptive immune response and *T. cruzi* infection represents a well-documented example of a systemic infectious process. Based on this, in the present study we have performed a differential blood cell counts to evaluate different changes in peripheral blood caused by MT and BT forms. Our major findings are that there is a late increase of all lymphocytes in the peripheral blood of the MT group, whereas in animals infected by BT forms an early and late increase (at day 7 and 42 after infection) of these cells occurs. These data show that infections with MT and BT forms lead to distinct immunological profiles. We hypothesized that as MT form requires an additional adjustment to the mammalian host, the infection starts with a silent onset and the massive recruitment of leukocytes will occur later, parallel with the peak of parasitemia. On the other hand, BT forms are more adapted to the mammalian host and therefore present a distinct infective profile that leads to an immediate invasion of host cells and thus lead to an earlier multiplication of the parasites, as seen in the parasitemia curve, as well as earlier changes in blood leukocytes.

Interestingly, only the animals in the MT group showed an increase of eosinophils during the infection. Nakhle *et al.* (1989) [22] infected mice, A/Sn (susceptible) and C57BL/6 (resistant) with the Y strain of *T. cruzi*, and noted a decrease in eosinophils in bone marrow and the peripheral blood in A/Sn animals, which did not occur in C57BL/6 mice, suggesting a greater capacity for eosinophilopoiesis after infection in C57BL/6 mice that might contribute to its greater resistance to infection. Moreover, Rowland & Sibley-Phillips (1984) [23] found that the largest number of eosinophils in the bone marrow of C57BL/6 coincided with the parasitemia peak at 28 days after infection. Based on these results, we infer that the lower parasitemia found in animals infected with the MT forms could be related to the increase of eosinophils in the peripheral blood, which would help in anti-trypanosomal activity. It is noteworthy that in the BT group were not found eosinophils during the acute phase similar to that observed in uninfected animals also accompanied by the same period (data not shown).

Despite the importance of eosinophils have been demonstrated in 80 years, there have been few recent studies involving the participation of these cells in Chagas disease. Nascentes *et al.* (2010) [24] to assess the participation of eosinophils in tissue damage in mice infected by VIC and JG strains of *T. cruzi*, also observed a higher number of eosinophils coincided with parasitemia peak in both strains used. Moreover, the number of eosinophils in the heart tissue was lower than in the skeletal muscle tissue. In this study, although it was observed an increase of eosinophils in the MT group, these cells were not observed in the inflammatory foci of the heart of these animals. So, apparently these cells are related to control of parasitemia.

*T. cruzi* infection promotes splenomegaly in mice and humans, and splenocytes are important cells involved in the host immune response. It has been shown that splenectomy prior to infection increase susceptibility to infection [25]. Furthermore, Sathler-Avelar *et al.* (2003) [26] and Kroll-Palhares *et al.* (2008) [27] reported that *T. cruzi* infection induces in both humans and experimental models important alteration in the host cellular immune response that reflect major phenotypic changes not only in the innate compartment, but also in the adaptive immunity context. Based on these facts, we carried out a phenotypic analysis of peripheral blood and spleen T lymphocytes. Our major findings in the blood demonstrate that there was a decrease in CD4^+^ and CD8^+^ T cells on day 7 after infection in BT-infected animals (data not shown). We hypothesize that this decrease reflects the recruitment for target tissues, which is in agreement with our results on cardiac inflammation that demonstrate an early migration of mononuclear cells in these animals. Padilla *et al.* (2009) [28] favor the hypothesis that the initial infection by *T. cruzi* is silent and that triggering of innate, and subsequently, adaptive immune responses does not occur until the first round of parasite replication and reinvasion (at 4–5 days post-infection) is complete. This is true for BT forms infection; however, infection by the MT forms does not affect the T-cell subpopulations in peripheral blood.

We have shown that, independent of the infective form, there is an increase in the percentage of CD8^+^ T cells in the spleen of mice infected with both trypomastigotes forms. Previous results from other workers have shown that CD8^+^ T cells are the major cell population in the heart tissue of chronic cardiomyophatic chagasic patients [29]. In this way, we could speculate that, since there is proliferation only in the spleen of CD8^+^ T cells at the end of the acute-phase, these cells migrate to the heart, becoming the main cell type found in cardiac inflammatory foci.

Earlier interactions between the host cells and *T. cruzi* are critical for the control of parasitemia, and for establishing a cytokine-rich microenvironment that will direct subsequent development of regulatory and effector T-cell populations, known to be important for the onset of pathology during the chronic phase. In the infection with MT forms cytokine production by CD4^+^ and CD8^+^ led to an early production of IFN-γ and only later there was an increase in the production of TNF-α. The opposite was observed in the infection with BT forms, where initially there was an increase of CD4^+^ and CD8^+^ TNF-α, and only later of T-cells IFN-γ^+^. It is known that *T. cruzi* is a potent inducer of TNF-α production by spleen cells *in vitro* [30]. This cytokine have a beneficial role early in infection, but also has harmful effects at the stage of patent parasitemia [31]. Animals infected with BT forms showed high early production (7^th^ day after infection) of this cytokine, which resulted in an overall balance of cytokines in inflammatory profile [32]. assessed the participation of TNF-α on cellular necrosis and destruction of infected macrophages in the spleen of mice during the acute phase of *T. cruzi* infection, they observed the expression of this cytokine in necrotic areas within the germinal center and in the red pulp, suggesting a role for TNF-α in the exacerbation of tissue damage. This same result was also observed recently by Andrade *et al.* (2008) [33]. Furthermore, kinetic investigations on the activation of macrophages during Chagas disease, demonstrated that macrophages of mice susceptible to infection produce higher levels of TNF-α than macrophages from resistant mice strains [34] [35]. It can be inferred then, that the high early production of TNF-α observed in BT group is associated with the exacerbation of the inflammatory process occuring in this group and could lead to higher cardiac injury. In addition, CD4 T lymphocytes appear to be induced to produce TNF-α, instead of IFN-γ on animals infected by BT forms.

Several studies have demonstrated that the cytokine IFN-γ has a protective effect on experimental infection with *T. cruzi*, *in vivo*, leading to macrophage activation by preventing immune system suppression and death of these animals during the acute phase [36]. Unlike observed for the animals in the BT group, the animals infected by a MT forms showed early production of IFN-γ, mainly by CD8^+^ cells. Because of this, these animals had an inflammatory profile that became immunoregulatory at the end of the acute phase, when the patent parasitemia was already controlled. These data coincide with the cardiac inflammation, which has its peak parallel to parasitemia peak, however when the parasitemia is already under control, it decreases so that it is a lack of inflammation in the animals of this group.

In this sense, animals that were infected by MT forms present an inflammatory status at the beginning of infection, but were able to induce an immunoregulatory response in spleen that is accompanied by a decreased in cardiac inflammation at the end of the acute phase, when the parasitemia has already come under control. Moreover, animals in the BT group failed to present an inflammatory response in early infection, which could be related to the earlier parasitemia observed in this group, and although they production a higher levels of IL-10 at the end of the acute phase, there is an exacerbation of the heart inflammatory process in this group.

The main goal of this investigation was to provide scientific data that MT and BT trigger distinct impact in peripheral blood, spleen and heart pool of inflammatory cells. It is important to mention that the interpretation of data generated in experimental models should be taken with caution since they may not reflect the complex parasite-host interaction observed in humans. Moreover, as the peripheral blood and spleen compartments do not decode the tissue compartmentalized immune response, further investigation in the heart tissue to characterize the cardiac pathology is necessary.

Our results reveal that MT and BT infections are associated with distinct cytokine profile during the acute phase of Chagas disease. In summary, our findings emphasize the importance of taking into account the inoculums source of *T. cruzi*, since vectorial or transfusional routes of *T. cruzi* infection may trigger distinct parasite-host interactions during the acute phase that may influence relevant biological aspects of chronic Chagas disease.

## Materials and Methods

### Ethics Statement

Details of the project were submitted and approved by the Ethical Committee on Animal Research of the Universidade Federal de Ouro Preto (approval ID number 2008/12). All procedures were carried out in compliance with current Brazilian Regulations relating to Experimental Biology and Medicine as described in the guidelines issued by the Colégio Brasileiro de Experimentação Animal (COBEA, 2006). Experimental animals were maintained in the central animal facility at the Universidade Federal de Ouro Preto (UFOP), Minas Gerais, Brazil.

### Parasites, animals and experimental infection

Nymphs of *Triatoma infestans* were allowed to feed on the blood of female Swiss mice (weight range 20–24 g) that had previously been inoculated with *T. cruzi* strain Be-78. Following infection, the triatomines were maintained under starvation conditions for 15 days and then allowed to blood-feed on uninfected mice in order to induce the release of metacyclic forms (MT) of the parasite in the faeces. Blood forms (BT) forms of the protozoan were obtained by infecting female Swiss Webster mice with 5×10^4^ blood forms of *T. cruzi* strain Be-78 per animal and collecting blood samples from the orbital veins at the parasitemia peak. After obtaining the both trypomastigotes forms, two groups of 30-day Swiss mice (30 g) were inoculated intraperitoneally with either MT or BT forms of Berenice-78 *T. cruzi* strain (5000 forms).

### Parasitemia and mortality parameters

Parasitemia of 5 Swiss mice from each group were determined daily under the optical microscope according to Brener [15]. Parasitemia curves were plotted using the daily mean numbers of parasites per group. Mortality was also observed daily. The experiment was performed in duplicate.

### Blood puncture

Blood from 5 animals in each group was collected by orbital plexus puncture in the period before infection (0) and at 7, 14, 28 and 42 days after infection to perform blood cell counts and to immunophenotype the cells by flowcytometry. This experiment was also conducted in duplicate.

### Mouse blood cell counts

The blood cell counts were determined using an electronic veterinary hematology particle counter from Mindray (BC-2800VET). The differential leukocyte count was perfomed by Giemsa-stained blood smears, counting a total of 100 cells per specimen by microscopy.

### Histopathological examinations

Experimental animals were examined by necropsy before infection (0) and at 7, 14, 28 and 42 days after infection. The heart was fixed in 10% buffered formalin (pH 7.2), and embedded in paraffin. Sections (5 µm thick) were mounted on glass slides and stained with Haematoxylin-Eosin (HE) for standard histological procedures.

### Determination of tissue parasitism

Embedded tissue sections were incubated overnight at 4°C with rabbit polyclonal anti-*T. cruzi* serum (obtained from *T. cruzi* Y strain immunized rabbit) diluted 1∶1000 in PBS. Subsequently, sections were incubated with secondary antibody anti-rabbit IgG and peroxidase – anti-peroxidase complex, and the label was detected by incubation with DAB. Sections obtained from canine acute myocarditis, that were rich in amastigote nests, were used as positive controls. Finally the sections were stained for nuclei with diluted Harris's haematoxylin solution. As negative controls the primary rabbit anti-*T. cruzi* serum was substituted by normal rabbit serum also diluted in PBS.

### Morphometric studies

Morphometric studies of inflammation involved analyzing images of 20 randomly-selected fields (total area 1.5×10^6^ µm^2^) of tissue sections on a single slide per animal. Inflammatory infiltration in the heart was quantified by counting the cell nuclei present in the sections of the hearts. *T. cruzi* immunoreactive areas were measured in sections of heart fragments. Images taken with a 40× objective were analysed with Leica QWin software (Leica Microsystems, Wetzlar, Germany).

### Flow cytometry immunophenotyping of blood cells

FITC labelled mAbs anti-CD4 or CD8 cells (Caltag, Burlingame, CA, USA) were put into polystyrene tubes. To each tube was added aliquots of whole peripheral blood collected in EDTA. After homogenization in a vortex, the preparations were incubated for 30 min at room temperature in the dark. After lysis of erythrocytes, the samples were centrifuged at 600 g for 7 min at room temperature. The supernatant was discarted and the leucocytes washed with 3 mL of PBS (pH 7.4), using the same centrifugation conditions as above. In the final step, the leukocytes were fixed with 200 µL in FACS FIX solution and stored at 4°C prior to flow cytometric acquisition and analysis. Phenotypic and morphometric parameters of cells present in each tube were determined by flow cytometry (FACScalibur ® - Becton Dickinson). The program CELLQuest® (Franklin Lakes, NJ, USA) was used for data acquisition and analysis of results from 10,000 events/sample.

### Spleen cell suspension and *in vitro* short-term culture of spleen cells

Five animals from each group were euthanized just before infection (0) and at days 7, 14, 28 and 42 after infection. The spleen was removed and cell suspensions prepared as described by Taylor *et al.* (1987) [16]. The organ was immersed in cold RPMI 1640 (5 mL) in a Petri dish and placed on ice for maceration. Fragments were squashed using a blunt glass rod and filtered through a stainless steel gauze to obtain a single-cell suspension. The suspension was washed twice in RPMI-1640 and resuspended at 1×10^7^cells/mL. Suspensions of spleen cells were incubated in the presence of 2 mL RPMI-1640 (GIBCO, Grand Island, NY, USA) in polypropylene tubes (Falcon, BD Pharmingen) for 12 h at 37°C in a 5% CO_2_ in air humidified incubator, followed by incubation with Brefeldin A (BFA) (Sigma, St Louis, MO, USA), at 10 µg/ml for an additional period of 4 h.

### Immunophenotyping of spleen cell subsets and intracellular cytokines

At the end of incubation period, the cultures previously treated with 2 mM EDTA (Sigma) were washed once with FACS buffer prepared as PBS with 0.5% bovine serum albumin and 0.1% sodium azide (Sigma). After resuspension in 2 ml of FACS buffer, 400 µl aliquots of suspension culture were immunostained with FITC labelled mAbs anti-CD4 or CD8 (Caltag, Burlingame, CA, USA) in the dark for 30 min at room temperature. After the lysing/fixation procedure, membrane-stained leukocytes were permeabilized with FACS perm-buffer (FACS buffer with 0.5% saponin) and incubated for 30 min at room temperature in the dark in the presence of 20 µl of PE-labelled anti-cytokine mAbs (IFN-γ, TNF-α and IL-10) from Serotec and Caltag, respectively. After intracytoplasmatic cytokine staining, the cells were washed and fixed in FACS FIX solution for storage at 4°C prior to flowcytometric acquisition and analysis. Immunostained samples were run in a FACScalibur® flow cytometer equipped with a 4-colour detection system (Becton Dickinson, San Jose, CA, USA). Data acquisition and analysis were done with CELLQUEST software (Franklin Lakes, NJ, USA) based on 30,000 events/sample. Unspecific binding was monitored by using fluorochrome-labeled isotypic matched reagents to provide confident negative controls. Autofluorescence was monitored by the use of a negative control were the cell suspension was incubated in the absence of fluorochrome-labeled mAbs, but in the presence of dilution and wash buffers. Flow cytometry compensation was carried out by previous instrument settings using calibration bead (CaliBrite® - Becton Dickinson, San Jose, CA, USA).

The lymphocytes were selected based on their relative flow cytometry size (forward laser scatter – FSC) and granularity (Side laser scatter – SSC). After flow cytometric instrument adjustments and settings, the lymphocyte population assumes a homogeneous distribution on FSC versus SSC dot plots and can be select as FSC^Low^ (<channel 200) and SSC^Low^ (<channel 200) events.

### Statistical analysis

Statistical analyses of the data were carried out using GraphPad Prism software 5.0 (San Diego, CA, USA). Data were assessed by one-way analysis of variance (ANOVA) between days; when interactions were significant, the Tukey test was used to determine the specific differences between mean values. Values have been expressed as means ± standard deviation, differences in mean values being considered significant at p<0.05.
